# Effect of Pre-Existing Micro-Defects on Cutting Force and Machined Surface Quality Involved in the Ball-End Milling Repairing of Flawed KDP Crystal Surfaces

**DOI:** 10.3390/ma15217407

**Published:** 2022-10-22

**Authors:** Hongqin Lei, Jian Cheng, Dinghuai Yang, Linjie Zhao, Mingjun Chen, Jinghe Wang, Qi Liu, Wenyu Ding, Guang Chen

**Affiliations:** 1 State Key Laboratory of Robotics and System, Harbin Institute of Technology, Harbin 150001, China; 2 School of Mechatronics Engineering, Harbin Institute of Technology, Harbin 150001, China

**Keywords:** KDP crystal, micro-milling repair, micro-defects, cutting force, machined surface quality

## Abstract

When serving in extremely high-power laser conditions, KH_2_PO_4_ (KDP) surfaces are susceptible to incur laser damage points (also known as defects). Using micro-ball end milling cutters to repair and remove the pre-existing damage points on the flawed KDP crystal surface is the most effective method to control the growth of laser damage points on KDP crystal surfaces and prolong their service life. However, there are various forms of micro-defects (such as pits, scratches and brittle fractures) around the laser damage points on KDP crystal surfaces which possess remarkable effects on the micro-milling repair process and consequently deteriorate the repair quality. In this work, combined with nano-indentation experiments, elastic–plastic mechanics and fracture mechanics theory, a constitutive model considering the anisotropic property of KDP crystals and a three-dimensional (3D) finite element model (FEM) were established to simulate the cutting force and surface topography involved in the ball-end milling repairing of flawed KDP crystal surfaces. Besides, the micro-milling experiments were conducted to evaluate the change of cutting force and machined surface quality in the presence of micro-defects with various feed rates. The results show that micro-defects would induce the fluctuation of cutting force and a change of the undeformed cutting thickness (UCT) in the process of repairing the damage points on the crystal surface, which would lead to the brittle–ductile transition (BDT) and affect the machined surface quality. The machined surface quality was found to be deteriorated by the pre-existing micro-defects when the UCT was small (the UCT was less than 375 nm). On the contrary, brittle mode cutting in the local area can be transformed into ductile mode cutting, resulting in an improvement of repaired surface quality that is exhibited by the cutting force and microtopography. This work has great theoretical significance and engineering practical value for the promotion and application of micro-milling repairing technology in the practical manufacturing and operation of KDP optics applied to high-power laser systems.

## 1. Introduction

With the growth of population and the development of productive social forces, the consumption of energy has increased dramatically [1,2]. However, due to the decrease of fossil energy and serious environmental pollution, countries around the world are engaged in the research and development of clean energy, such as biofuels and nuclear energy [3,4]. At present, inertial confinement fusion (ICF) is considered to be the ultimate solution to the human energy crisis because of its safety, controllability, environmental protection and renewable advantages. Due to its excellent photoelectric characteristics [5,6], the KDP crystal has become an irreplaceable large-scale frequency doubling element, and photoelectric switching has been extensively applied to laser-driven ICF facilities. However, high-power laser irradiation is prone to inducing different types of damage points on the crystal surface [7]. These damage points rapidly expand in the subsequent laser-shooting process, which seriously affect the optical performance and service life of KDP crystal components [8]. Previous research has shown that repairing the initial damage points on the crystal surface with micro ball-end milling is the most effective method to control the growth behavior of pre-existing laser damage points on the flawed surfaces of KDP crystal components and prolong their service life [9]. However, due to the low hardness, high brittleness, incidental deliquescence, remarkable anisotropy and high processing accuracy requirements, the urgent demand for large-scale, high-precision and low-defect-density KDP crystal components poses an unprecedented challenge in the ultra-precision optical processing and manufacturing fields [10].

Achieving ductile mode cutting of brittle materials can obtain smooth surfaces [11,12]. Previous studies mainly include the brittle–ductile transition (BDT) mechanism, surface quality prediction, and chip morphologies in different cutting modes. Research methods were composed of experimental research methods (nanoindentation and microgroove experiments) and numerical simulation methods (molecular dynamics and constitutive model establishment). Yan et al. [13] investigated the micromachining processes of the BDT of different single crystal germanium surfaces, and obtained the critical cutting depth for BDT (BDd_c_) according to the chip morphologies with different cutting thicknesses. Malekian et al. [14] proposed an analytical model by identifying the critical points of workpiece material processing. It showed that the minimum UCT is not only a function of the edge radius and friction coefficient, but also depends on the tool geometry and workpiece material characteristics. Arif et al. [15] used the mathematical model to determine the critical conditions for milling on the crack-free surface of brittle materials, and the experimental results verified the proposed processing model. Liang et al. [16] studied the BDT mechanism of single-crystal sapphire through micro-groove machining. Zhou et al. [17] established the energy ratio consumed by the removal of the ductile and brittle modes as a function of the grinding parameters and the inherent characteristics of the workpiece materials, and successfully verified the effectiveness of the model. Gu et al. [18] studied the effects of geometrical parameters of cutting tools on the BDT of sapphire during the machining process by micro-groove machining. Chen et al. [19] obtained the BDd_c_ of [110] the crystal orientation of gallium arsenide through micro-groove machining with variable cutting depth. Liu et al. [20] proposed a theoretical mathematical model to predict the d_c_, through which the d_c_ of Si_3_N_4_ ceramics was calculated to be 0.38 mm. Besides, the theoretical model showed that d_c_ decreased exponentially with the increase of material hardness. Tie et al. [21] studied the BDd_c_ of KDP crystals in different cutting directions by spiral scratch, and obtained an ultra-smooth machined surface. Gouskou et al. [22] installed a vacuum suction chip device on a Toshiba ultra-precision machine tool. An ultra-smooth surface with a roughness of *Ra* = 1.6 nm was processed, and the maximum UCT of KDP crystals was 17 nm. Although the abovementioned work is mainly focused on achieving ductile mode cutting for the defect-free surface of brittle materials, establishing the UCT models, and exploring the influence of the tool parameters or process parameters on the BDT, there are few efforts spent on ductile mode cutting on flawed KDP crystal surfaces. Thus, it is of great significance to explore the effect of micro-defects on the BDT and machined surface quality in the ball-end milling repairing of KDP crystals.

In addition, the finite element model (FEM) was gradually applied to simulate the cutting process. The simulated results of the cutting force and machined surface topography could effectively predict the machined surface quality and explore the ductile cutting process parameters. Lee et al. [23] applied a crystal constitutive equation to the FEM of the microscale chip formation, and predicted that the change of the cutting force was in good agreement with the experimental results. Cao et al. [24] used a two-dimensional (2D) orthogonal model to simulate the cutting process of KDP crystals, and found that the BDd_c_ was about 140 nm. The relative error between the simulated result and the critical cutting thickness measured by the experiment was less than 10%. Xiao et al. [25] studied the mechanism of BDT, proposed a mechanical model, and analyzed the relationship between the UCT, deformation and stress level in the elastic stage of the periodic chip formation process. Chen et al. [26] proposed a new method to model and simulate the surface formation of KDP crystals in the process of fly-cutting at the macroscales, microscales and nanoscales, in order to establish evaluation criteria of different spatial frequency regions. In the molecular dynamics simulation of the nano-cutting process, Chen et al. [27] found that the damage type changed from the line defect to the phase transition with the increase of cutting speed. Besides, when the number of cracks increased with greater cutting depth, the deformation mode changed from ductility to a combination of ductility and brittleness. Chen et al. [28] established a 3D simulation model of the micro-milling repair process for KDP crystals by analyzing the key issues, such as workpiece separation criteria and material parameters. The simulated results were consistent with that in the experiment. Wang et al. [29] transformed the constitutive model that could describe the mechanical behavior of KDP crystals into the finite element code in the LS-DYNA, which was found to be accurate to reproduce the phenomenon of the anisotropic cutting force. It could be summarized that, in terms of simulations of the cutting process for brittle materials, the micro-milling simulation model is usually simplified to a 2D orthogonal cutting model. Not only does it not consider the influence of the real shape of the milling cutter, but also the simulated results cannot be reflected from three dimensions. More importantly, a constitutive model suitable for describing the material properties of KDP crystals has not been established, which seriously reduces the authenticity of the micro-milling simulations.

In the engineering applications of KDP optics, there are many kinds of micro-defects, such as pits and scratches, around the laser damage points on the flawed KDP surfaces. However, the fluctuation of cutting force and the change of UCT induced by these pre-existing micro-defects would affect the machined surface quality of the crystal components, and finally affect the laser damage resistance of the repaired KDP surfaces in the process of repairing the damaged points on the crystal surface. Therefore, exploring the influence law of the pre-existing micro-defects around the laser damage points on the mechanical properties of KDP crystals and revealing the influence mechanism of micro-defects in the micro-milling repair process have important theoretical significance and engineering practical value for the promotion and application of micro-milling repairing technology in the ordinary operation and maintenance of laser-driven ICF facilities.

In this work, as one of the most common micro-defects on the fly-cutting surface of KDP crystals, the scratch defect could change the UCT in the ball-end milling repairing and further affect machined surface quality. Therefore, it was taken as an example to explore the effect of pre-existing micro-defects on the flawed KDP crystal surface on repaired surface quality. Based on a proposed constitutive model considering the anisotropic property of KDP crystals, the 3D simulation model of the micro-milling repair process was established in Section 2. The details of the experimental equipment and process were described in Section 3. Then, the influence of pre-existing micro-defects on cutting force and machined surface quality were discussed in Section 4. Finally, the conclusion was drawn in Section 5.

## 2. Finite Element Modeling and Simulation

### 2.1. Three-Dimensional Modeling of Micro-Milling Repair Process for KDP Crystals

**Figure 1:**
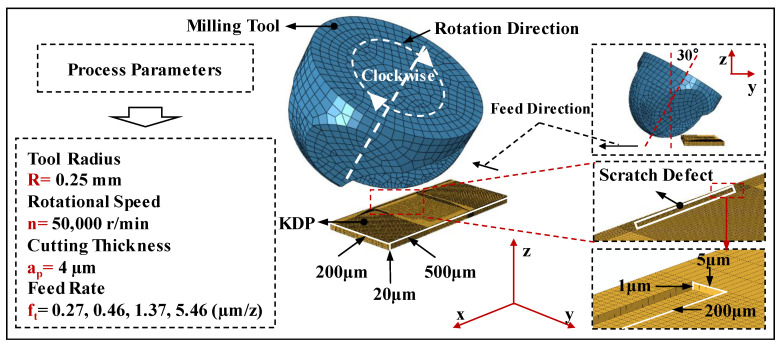
The schematic of the established 3D simulation model of KDP micro-milling process.

Figure 1 shows the 3D simulation model of the micro-milling repair process for KDP crystals, which is composed of the micro-ball end milling cutter and the KDP crystal. The geometric model of the ball-end milling cutter with a complex shape was established by computational-aided design (CAD). To greatly reduce the calculation cost, only the cutter head was meshed because it is actually involved in the micro-milling repairing of KDP crystals. Meanwhile, since the hardness of the tool material is far greater than that of the KDP crystals, a discrete rigid body was adopted to model the tool with the tetrahedral-element mesh. In contrast, a deformable model was applied to the KDP crystal elements with 500 μm length, 200 μm width and 20 μm height. The scratch defect was located on the crystal component surface and the length, width and depth were 200 μm, 5 μm and 1 μm, respectively. The mesh of the processed material was set as hexahedral elements considering the shape of the scratch defect. In order to save calculation time, the local mesh subdivision method was adopted to subdivide the part of the KDP crystals participating in the interaction process with the end of the ball-milling cutter. The total number of meshes was about 120,000, and the calculation time was roughly 2 days (2 × 24 h). Based on the actual processing parameters of ball-end milling repairing for KDP crystals, the ball-end milling cutter was inclined by an angle of 30° in the model to avoid the part with zero linear velocity participating in the micro-milling process. The feed rate was set to 1 mm/s along the Y direction. The cutting depth in the Z direction was set to 2 μm. The rotation speed of the spindle was 50,000 r/min, and it rotated around the axis of the tool in a clockwise direction.

### 2.2. The Establishment of KDP Crystal Constitutive Model Considering the Anisotropic Mechanical Property

According to the mechanical properties of KDP crystals, a constitutive model is proposed to describe anisotropic mechanical properties. In the elastic stage, the elastic stiffness matrix is used to describe the elastic properties of KDP crystals. In the plastic stage, the power hardening model is used to describe the stress–strain relationship of KDP crystals [30], and the yield stress ratio is introduced to characterize the anisotropy of its material properties. In the fracture stage, the maximum tensile strain failure criterion is adopted as the fracture criterion.

In this work, the stress–strain curve in the plastic domain of KDP crystal material was obtained through nanoindentation experiments. It is assumed that the stress–strain at the ideal yield point satisfies the stress–strain relationship at both the elastic and the plastic stage:

(1) $$\sigma_{0}=E\varepsilon_{0}$$

(2) $$\sigma_{0}=K\varepsilon_{0}{}^{\frac{1}{n}}$$

where *σ*_0_ is the yield stress, *E* is the elastic modulus, *ξ*_0_ is the yield strain, *K* is the plastic modulus, and *n* is the strain hardening index.

The elastic modulus *E* is calculated as follows:

(3) $$\frac{1}{E_{r}}=\frac{\left(1-v^{2}\right)}{E}+\frac{\left(1-v_{i}{}^{2}\right)}{E_{i}}$$

where *E*_r_ is the equivalent elastic modulus obtained from the unloading curve in the indentation experiment, *ν* is the Poisson’s ratio of KDP crystals, *ν_i_* is the Poisson’s ratio of indenter, and *E_i_* is the elastic modulus of indenter.

The simultaneous solution of Equations (1)–(3) shows that the stress–strain relationship of KDP crystals in the nanoindentation experiment consists of elastic and plastic stages, as shown in the following formula:

(4) $$\sigma =\{\begin{array}{ll} 33200\varepsilon & \left(\sigma \leq 360MPa\right)\\ 2375.1\varepsilon^{0.417} & \left(\sigma \geq 360MPa\right) \end{array}$$

where *σ* is the nominal stress and *ξ* is the nominal strain.

It is necessary to input the real stress and plastic strain in the cutting simulation model. The relationship between real stress and nominal stress/strain is as follows:

(5) $$\sigma_{true}=\sigma \left(1+\varepsilon \right)$$

where *σ*_*true*_ is the true stress.

The nominal strain calculated in the nanoindentation experiment is the total strain of the material, which includes the plastic strain and elastic strain. The expression of the relationship is as follows:

(6) $$\varepsilon_{pl}=\ln\left(1+\varepsilon \right)-\frac{\sigma_{true}}{E}$$

where *ξ_pl_* is the plastic strain.

Here, the HILL yield criterion is adopted to consider the anisotropy in the yield stage of KDP crystals, and the formula of equivalent stress is as follows:

(7) $$\sigma_{0}=\sqrt{F{\left(\sigma_{22}-\sigma_{33}\right)}^{2}+G{\left(\sigma_{33}-\sigma_{11}\right)}^{2}+H{\left(\sigma_{11}-\sigma_{22}\right)}^{2}+2L\sigma_{23}{}^{2}+2M\sigma_{31}{}^{2}+2N\sigma_{12}{}^{2}}$$

where *F*, *G*, *H*, *L*, *M* and *N* are constants obtained by testing the mechanical properties of KDP crystals in different directions; *σ*_11_, *σ*_22_ and *σ*_33_ are the normal stress; and *σ*_23_, *σ*_31_ and *σ*_12_ are the shear stress.

According to the constants *F*, *G*, *H*, *L*, *M* and *N* of HILL potential function, the solution equation of the yield stress ratio is as follows:

(8) $$F=\frac{1}{2}\left(\frac{1}{R_{22}{}^{2}}+\frac{1}{R_{33}{}^{2}}-\frac{1}{R_{11}{}^{2}}\right)$$

(9) $$G=\frac{1}{2}\left(\frac{1}{R_{33}{}^{2}}+\frac{1}{R_{11}{}^{2}}-\frac{1}{R_{22}{}^{2}}\right)$$

(10) $$H=\frac{1}{2}\left(\frac{1}{R_{11}{}^{2}}+\frac{1}{R_{22}{}^{2}}-\frac{1}{R_{33}{}^{2}}\right)$$

(11) $$L=\frac{3}{2R_{23}{}^{2}}$$

(12) $$M=\frac{3}{2R_{13}{}^{2}}$$

(13) $$N=\frac{3}{2R_{12}{}^{2}}$$

where *R*_11_, *R*_22_, *R*_33_, *R*_12_, *R*_13_ and *R*_23_ are the yield stress ratios.

*F*, *G*, *H*, *L*, *M* and *N* are obtained through the uniaxial tensile tests along the main axis of material anisotropy (x, y, z) and the shear tests in plane (yoz, xoz, xoy). The yield stress ratios can be calculated through Equations (8)–(13), as shown in Table 1.

The maximum fracture strain value is added in the material property settings because KDP crystals cannot follow the stress–strain relationship in the cutting process as in the nanoindentation experiment. The crystal would fracture, and the stress value would drop to zero when the ductile strain value of KDP crystals in the micro-milling process is greater than this critical value. The commonly used elastic stiffness matrix [c] can be expressed by the following formula [31]:

(14) $$\left[c\right]=\left[\begin{array}{cccccc} 7036 & -4830 & 13440 & 0 & 0 & 0\\ -4830 & 70360 & 13440 & 0 & 0 & 0\\ 13440 & 13440 & 56800 & 0 & 0 & 0\\ 0 & 0 & 0 & 12800 & 0 & 0\\ 0 & 0 & 0 & 0 & 12800 & 0\\ 0 & 0 & 0 & 0 & 0 & 12800 \end{array}\right]$$

The material properties of KDP crystals are shown in Table 2 [32]. The geometric parameters of the ball-end milling cutter are shown in Table 3. The tool material used was cubic boron nitride (CBN), and its material properties are shown in Table 4 [33,34].

## 3. Experimental Procedure

**Figure 2:**
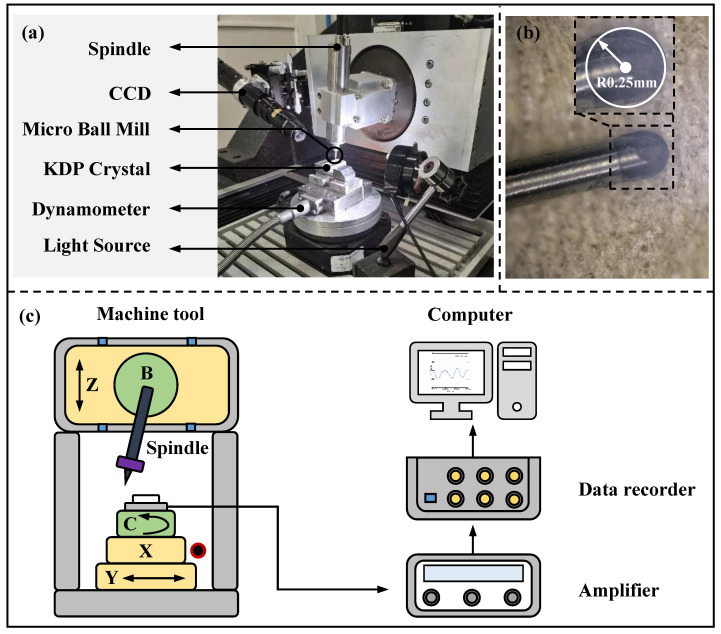
The schematic of the micro-milling device: (**a**) experimental device for micro-milling KDP crystals; (**b**) the geometry of the ball-end milling cutter; (**c**) the experimental setup for measurement of cutting force.

The micro-milling repairing experiments for KDP crystals were conducted on the self-developed five-axis milling CNC machine tool, and the radius of the ball-end milling cutter was 0.25 mm, as shown in Figure 2. The cutting force signal was collected through the dynamometer (KISTLER 5197A, Kistler, Winterthur, Switzerland) in the cutting process. Additionally, the machined surface was observed by an ultra-depth-of-3D-field microscope (VHX 1000E, Keyence, Osaka, Japan) and the 3D morphological characteristics of the machined surface were measured by a white light interferometer (ZYGO 8200, Zygo Corporation, Middlefield, CT, USA). The external dimensions of the machine tool were 1200 mm × 900 mm × 1750 mm. The maximum speed of its air-bearing spindle could reach 80,000 rpm, and the runout was less than 1 µm. The straightness positional precision could reach 0.5 µm/50 mm.

**Figure 3:**
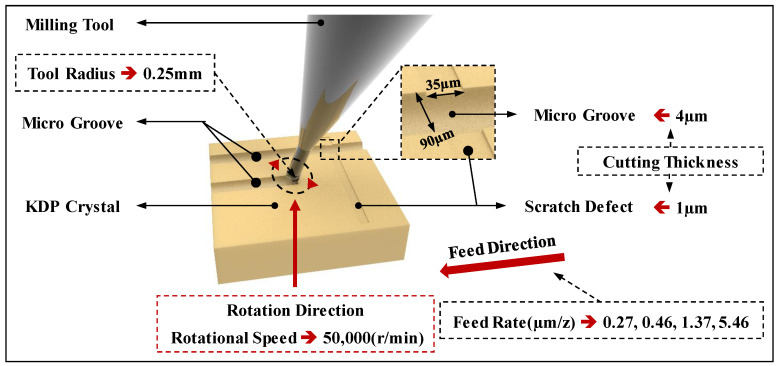
The schematic of the micro-milling repair process for flawed KDP crystal surfaces.

Before the micro-milling experiments, a MSB230 ball-end milling cutter (NS TOOL Co., Ltd., Shinagawa, Japan) with a radius of 0.15 mm was adopted to prepare a scratch defect with a depth of 1 µm and width of 35 µm on the KDP crystal surface. A SSBL200 ball-end milling cutter (NS TOOL Co., Ltd.) with a radius of 0.25 mm was adopted to process the microgroove, making it pass through the scratch defect, as shown in Figure 3. In this way, the experimental procedure can be used to explore the effect of pre-existing micro-defects on cutting force and machined surface quality in the micro-milling repair process of flawed KDP crystal surfaces. By setting the control group, the results of cutting force and machined surface quality are compared and analyzed. The adopted cutting parameters are listed in Table 5. For each group of experiments, a 20 mm long microgroove was processed and the experiment was repeated three times in the same conditions to guarantee that the error of the experimental results was as small as possible.

## 4. Results and Discussion

### 4.1. Cutting Force and Machined Surface Quality in the Case of the Defect-Free Surface

**Figure 4:**
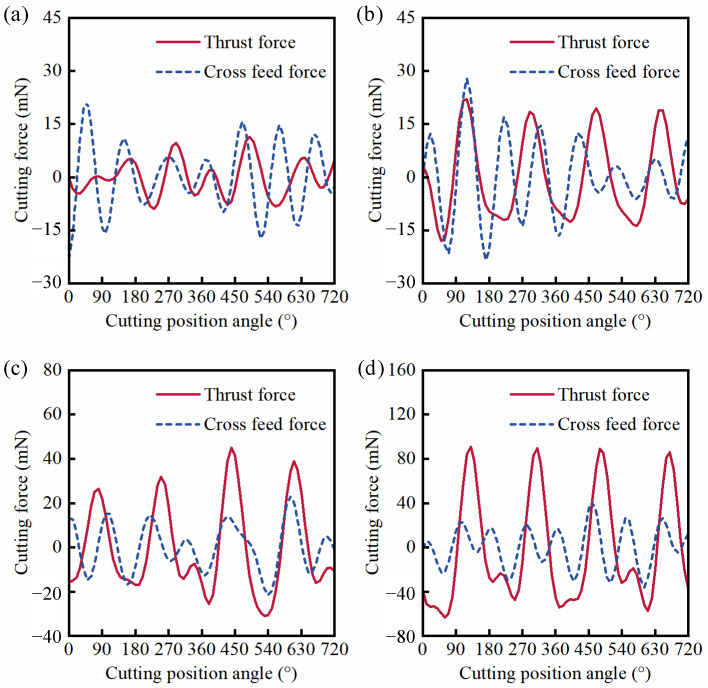
(Defect-free surface) Experimental results of cutting force with cutting edge rotation angle under *f*_t_/*r*_e_: (**a**) *f*_t_ = 0.27 µm/z, *f*_t_/*r*_e_ = 0.15; (**b**) *f*_t_ = 0.46 µm/z, *f*_t_/*r*_e_ = 0.25; (**c**) *f*_t_ = 1.37 µm/z, *f*_t_/*r*_e_ = 0.75; (**d**) *f*_t_ = 5.46 µm/z, *f*_t_/*r*_e_ = 3.0.

In order to contrast and analyze the effect of pre-existing scratch defects on cutting forces and machined surface quality, firstly, this section briefly describes the change of the cutting force and machined surface quality of KDP crystals in the micro-milling repair process for the defect-free surface. The cutting force can directly reflect the interaction between the tool and the workpiece material in the cutting process. Considering the small amplitude and irregular fluctuation of feed force in the micro-milling process, only axial and transverse feed forces are discussed and analyzed in this work. The cutting force has four peaks and troughs during two turns of tool rotation in theory because the milling cutter has two cutting edges. The cutting force signal of the defect-free machined surface is shown in Figure 4 under different feed rates. It can be seen from Figure 4a that the cutting force has serious relaxation and oscillation under a feed rate of 0.27 µm/z, which is no longer approximate to the sinusoidal function, indicating that the ploughing effect is very serious [35]. In Figure 4b, there are four peaks and troughs of the thrust force within two entire cutting circles of 720° under a feed rate of 0.46 µm/z, but the cross feed force is still irregular, indicating that the ploughing effect has been greatly reduced and the machined surface quality is improved (Figure 4c). When the feed rate increases to 1.37 µm/z (the UCT is 375 nm), the cutting force signal curve has become relatively smooth, approximating a sine function, which proves that there is almost no ploughing effect in the machining process. However, Figure 4d shows the cutting force fluctuates in a zigzag shape when the feed rate increases to 5.46 µm/z, indicating that the brittle mode cutting has occurred. According to the above results of the cutting force, it can be seen that there are three cutting modes in the micro-milling process of KDP crystals due to the variation of UCT.

**Figure 5:**
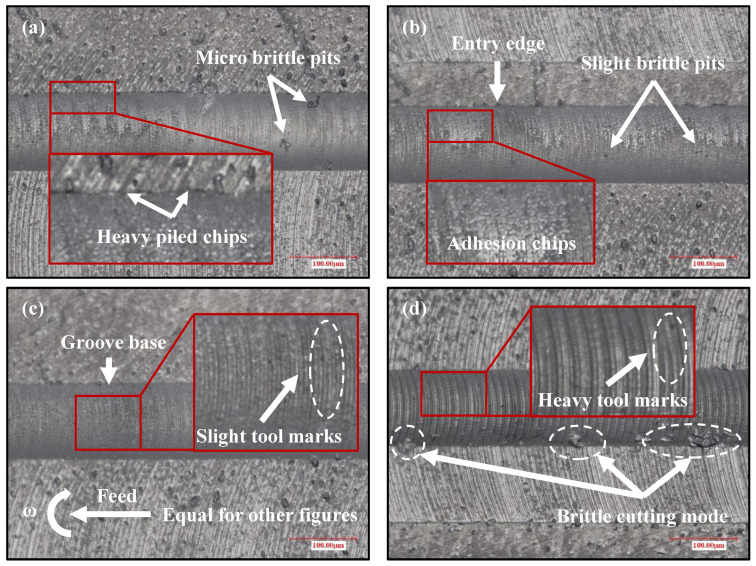
(Defect-free surface) Machined surface topographies of microgrooves with different feed rates per tooth: (**a**) *f*_t_ = 0.27 µm/z, *f*_t_/*r*_e_ = 0.15; (**b**) *f*_t_ = 0.46 µm/z, *f*_t_/*r*_e_ = 0.25; (**c**) *f*_t_ = 1.37 µm/z, *f*_t_/*r*_e_ = 0.75; (**d**) *f*_t_ = 5.46 µm/z, *f*_t_/*r*_e_ = 3.0.

In addition to studying the change of cutting force under different feed rates, further exploring the change of the surface topography can fully reveal the effect of process parameters on the machined surface quality. It can be seen from Figure 5a that there were micro brittle pits on the surface of the microgroove under a feed rate of 0.27 µm/z. Besides, the material accumulated on the cutting side was mainly chips that were broken and adhered to the crystal surface. This phenomenon confirms that the ploughing effect shown in Figure 4a is very serious [9]. Figure 5b shows that there are slight brittle pits at the bottom of the microgroove and chips adhered to its surface when the feed rate increases to 0.46 µm/z. The reason is that the size effect leads to the ploughing phenomenon in the cutting process. The cutting zone would be subject to excessive extrusion and ploughing when the UCT is much less than the radius of the cutting edge. Therefore, the crystal material is removed in the form of the brittle mode due to excessive tensile stress [36]. Meanwhile, the generated chips are easily embedded into the crystal surface with the rotation of the cutting edge to form material accumulation. Figure 5c shows that the machined surface quality of the microgroove is significantly improved under feed rate of 1.37 µm/z, leaving only clear tool marks at the bottom, without chip accumulation and brittle fracture points, indicating the characteristics of the ductile mode. It agrees well with the results in Figure 4c. A large number of brittle fracture points appear on the cut side of the microgroove, and the tool marks are more serious when the feed rate increases to 5.46 µm/z (Figure 5d). This indicates that the brittle mode cutting has occurred because the current UCT is larger than the BDd_c_, confirming that this phenomenon occurs under this feed rate as described in Figure 4d. From the results and discussion above, with the increase of feed rate, the UCT increases and the ploughing effect is weakened until the ductile mode occurs. However, when the UCT is larger than the BDd_c_, the brittle mode occurs in the ball-end milling repairing of defect-free surfaces [11].

**Figure 6:**
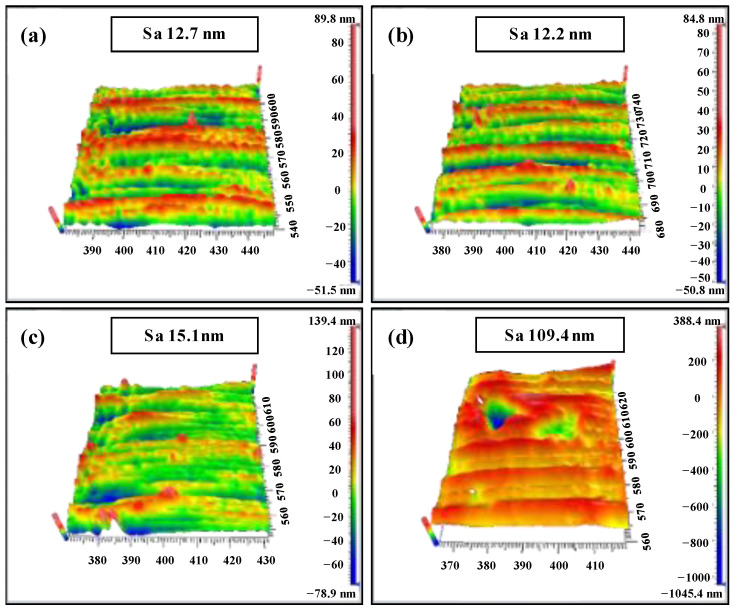
(Defect-free surface) Three-dimensional morphological characteristics of experimentally processed microgrooves with different feed rates per tooth: (**a**) *f*_t_ = 0.27 µm/z, *f*_t_/*r*_e_ = 0.15; (**b**) *f*_t_ = 0.46 µm/z, *f*_t_/*r*_e_ = 0.25; (**c**) *f*_t_ = 1.37 µm/z, *f*_t_/*r*_e_ = 0.75; (**d**) *f*_t_ = 5.46 µm/z, *f*_t_/*r*_e_ = 3.0.

In order to verify the variation of cutting force and surface topography under different feed rates, the white light interferometer was applied to measure the 3D morphology of the machined surface. It can be seen from Figure 6a,b that the machined surface has a large number of chips adhered to the microgroove surface under a feed rate of 0.27 µm/z, and the overall surface roughness is slightly higher than the machined surface under feed rate of 0.46 µm/z. It was confirmed that the ploughing effect under a feed rate of 0.27 µm/z is more serious than that under a feed rate of 0.46 µm/z. Compared with the machined surface quality under a feed rate of 1.37 µm/z (Figure 6c), there are deep brittle fracture points on the microgroove surface, and the surface roughness is as high as 109.4 nm, which shows that the machined surface quality is worse when the feed rate increases to 5.46 µm/z. The change of cutting force and machined surface quality under different feed speeds were studied and the crystal surface used in the experiment had no micro-defects in the above work. For comparison with the case of the defect-free surface, the following experiment explored the effect of the pre-existing micro-defect on the cutting force and machined surface quality of the scratched surface by the micro-milling simulation and experimental methods.

### 4.2. Effect of the Scratch Defect on the Cutting Force

**Figure 7:**
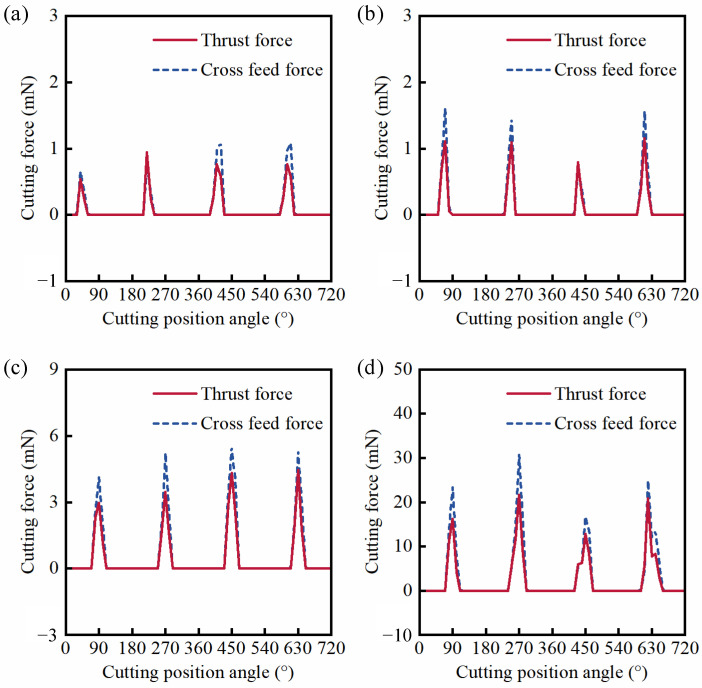
(Scratched surface) Simulated results of cutting force with cutting edge rotation angle under *f*_t_/*r*_e_: (**a**) *f*_t_ = 0.27 µm/z, *f*_t_/*r*_e_ = 0.15; (**b**) *f*_t_ = 0.46 µm/z, *f*_t_/*r*_e_ = 0.25; (**c**) *f*_t_ = 1.37 µm/z, *f*_t_/*r*_e_ = 0.75; (**d**) *f*_t_ = 5.46 µm/z, *f*_t_/*r*_e_ = 3.0.

The simulations of the cutting force on the scratched surface are shown in Figure 7. It can be seen that the cutting force curve appears with four peaks and valleys when the ball-end milling cutter with two edges rotates twice, which is in good agreement with the experimental results (Figure 4c). The cutting force gradually increases with the increase of the feed rate. Additionally, in the presence of a micro scratch defect, the cutting force of the material removal area becomes smaller because the UCT decreases due to the scratch defect. Compared with the simulated results in Figure 7b, the cutting force curve has more relaxation and oscillation in Figure 7a. The reason is that the UCT under a feed rate of 0.27 µm/z is not only smaller than that under a feed rate of 0.46 µm/z, but also is far less than the radius of the cutting edge. Therefore, a more serious ploughing effect is generated. When the feed rate increases to 1.37 µm/z, there is a slight relaxation and oscillation of the cutting force, as shown in Figure 7c. Figure 7d shows the cutting force appears to show a slight zigzag fluctuation when the feed rate increases to 5.46 µm/z, because the ductile mode cutting occurs in this process due to the UCT in the local area being less than the BDd_c_. The above simulations of cutting force show that the fluctuations of cutting force in the local area are induced by the micro scratch defect, indicating that the cutting mode is transformed simultaneously.

**Figure 8:**
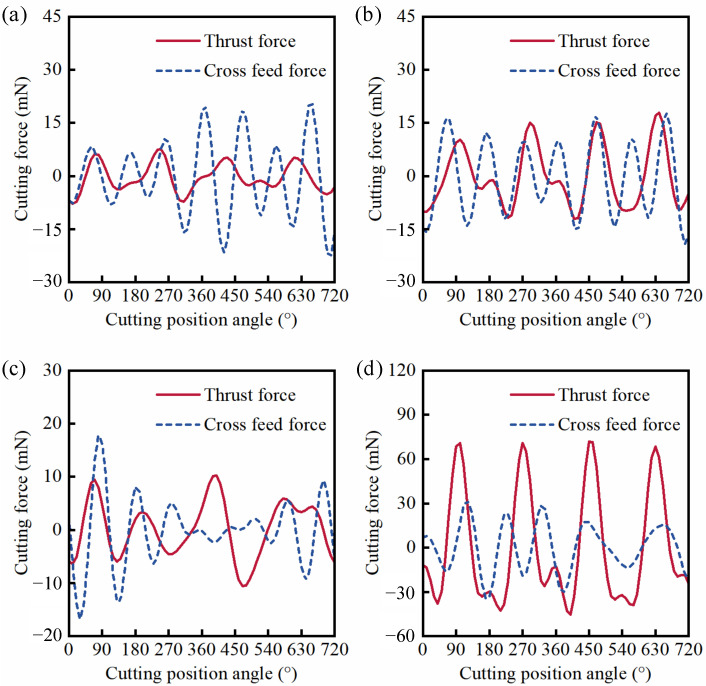
(Scratched surface) Experimental results of cutting force with cutting edge rotation angle under *f*_t_/*r*_e_: (**a**) *f*_t_ = 0.27 µm/z, *f*_t_/*r*_e_ = 0.15; (**b**) *f*_t_ = 0.46 µm/z, *f*_t_/*r*_e_ = 0.25; (**c**) *f*_t_ = 1.37 µm/z, *f*_t_/*r*_e_ = 0.75; (**d**) *f*_t_ = 5.46 µm/z, *f*_t_/*r*_e_ = 3.0.

Hence, to verify the simulations of cutting force above, the cutting force of flawed KDP crystal surfaces under different feed rates was measured by the dynamometer in the micro-milling repairing experiment, as shown in Figure 8. Compared with the results in Figure 4a,b, it can be seen from Figure 8a,b that the cutting force not only reduces, but also that there is more relaxation and oscillation, indicating that the micro scratch defect leads to more serious ploughing effects. When the feed rate increases to 1.37 µm/z (Figure 8c), compared with the results in Figure 4c, the cutting force shows a little relaxation and oscillation, which is no longer approximate to the sine function. It indicates that the ploughing effect occurs in the micro-milling repair process of KDP crystals. However, compared with the results in Figure 4d, although the cutting force reduces, the signal curve has become smoother with only a little serration when the feed rate increases to 5.46 µm/z (Figure 8d). From the results and discussion above, compared with the case of the defect-free surface at the same feed rate, the amplitude and fluctuation of the cutting force can be altered by the micro scratch defect in the micro-milling repair process of flawed KDP crystal surfaces.

### 4.3. Effect of the Scratch Defect on the Machined Surface Quality

**Figure 9:**
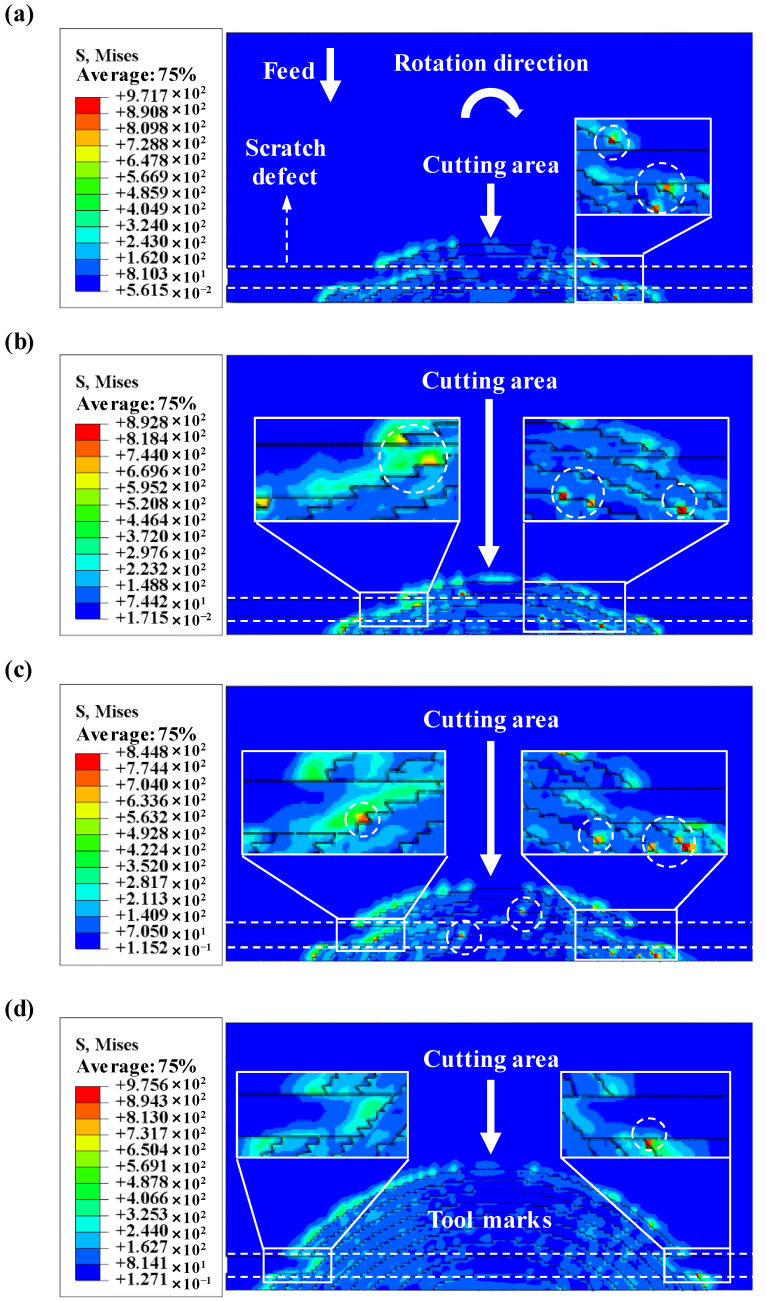
(Scratched surface) Simulated results of microgroove surface with different feed rates per tooth: (**a**) *f*_t_ = 0.27 µm/z, *f*_t_/*r*_e_ = 0.15; (**b**) *f*_t_ = 0.46 µm/z, *f*_t_/*r*_e_ = 0.25; (**c**) *f*_t_ = 1.37 µm/z, *f*_t_/*r*_e_ = 0.75; (**d**) *f*_t_ = 5.46 µm/z, *f*_t_/*r*_e_ = 3.0.

In order to comprehensively evaluate the effect of micro-defects on the machined surface quality, besides the cutting force, the effect of the micro scratch defect on the surface topography in the micro-milling repairing of flawed KDP crystals surfaces was further studied by the micro-milling simulation and experimental methods. Combined with the simulations in Figure 9b, the maximum tensile stress areas are densely distributed on the cut-out side at the scratch defect and it can be found from Figure 9a that the tensile stress on the scratched surface is higher. On the one hand, the size effect is more serious when the UCT under a feed rate of 0.27 µm/z is smaller than that under a feed rate of 0.46 µm/z. On the other hand, the scratch defect on the crystal surface results in a sudden change of the cross-section area. Therefore, there is a more serious stress concentration phenomenon near the defect edge [37]. It is confirmed that the scratch defect could result in a more serious ploughing effect as shown in Figure 8a,b. Although the tensile stress of the scratched surface reduces, the maximum tensile stress area appears at the scratch defect in the middle of the cutting area when the feed rate increases to 1.37 µm/z, as shown in Figure 9c. This indicates that the UCT reduces due to the scratch defect, so the ploughing effect occurs at the cutting area. It agrees well with the results in Figure 8c. However, although the tensile stress of the scratched surface is extremely high, the tensile stress in the middle of the cutting area is smaller when the feed rate increases to 5.46 µm/z (Figure 9d) because the scratch defect induces the UCT of this area to be smaller than the BDd_c_. Therefore, the machined surface quality of this area is interestingly found to be improved, and only a few maximum-tensile-stress concentration areas appear on the cut-out side. This phenomenon confirms that there is ductile mode cutting in the local area. Compared with the case of the scratch-free surface, the simulations of the machined surface topography show that the micro scratch defect can result in the transformation of the cutting mode in the local area and change the microtopography.

**Figure 10:**
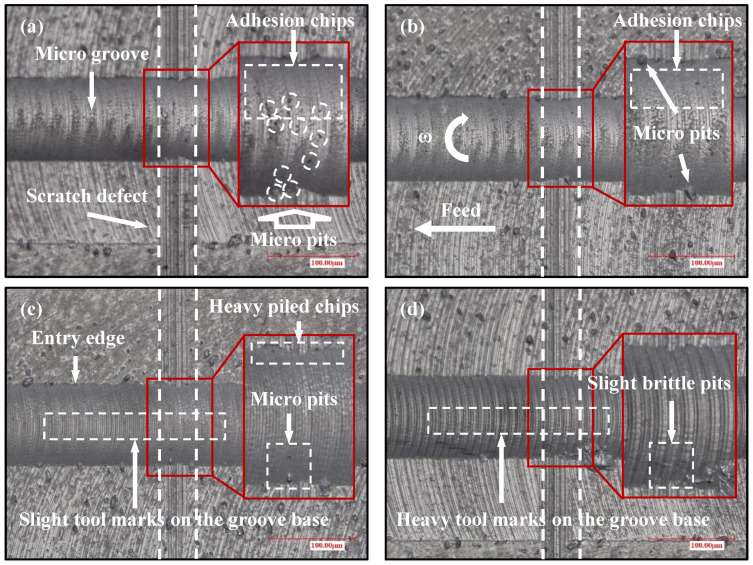
(Scratched surface) Machined surface topographies of microgroove with different feed rates per tooth: (**a**) *f*_t_ = 0.27 µm/z, *f*_t_/*r*_e_ = 0.15; (**b**) *f*_t_ = 0.46 µm/z, *f*_t_/*r*_e_ = 0.25; (**c**) *f*_t_ = 1.37 µm/z, *f*_t_/*r*_e_ = 0.75; (**d**) *f*_t_ = 5.46 µm/z, *f*_t_/*r*_e_ = 3.0.

To verify the simulated results of the surface topography, the machined surface topography of flawed KDP crystal surfaces under different feed rates was measured by the ultra-depth-of-3D-field microscope in the micro-milling repairing experiment, as shown in Figure 10. Compared with the results of Figure 5, it can be seen from Figure 10a that the number of micro pits on the surface of the microgroove and chips accumulated in the cutting side area both increase under a feed rate of 0.27 µm/z. In Figure 10b, it can be observed that micro pits of a large size are distributed on the cut-in and cut-out sides of the microgroove under a feed rate of 0.46 µm/z. Meanwhile, the chips adhered to the microgroove surface are denser. The reason is that the UCT reduces due to the scratch defect, which is much smaller than the radius of the cutting edge. Therefore, the stronger size effect results in a more serious ploughing, and leads to the deterioration of the machined surface quality. It is in good agreement with the simulated results in Figure 9a,b. Due to the scratch defect, the reduction of UCT results in a more serious ploughing effect. When the feed rate increases to 1.37 µm/z, Figure 10c shows that there are not only serious chip accumulations on the cut-in side, but also brittle fracture points on the cut-out side. This indicates that the ploughing effect occurs in the micro-milling repair process of KDP crystals, agreeing well with the simulated results in Figure 9c. However, owing to the scratch defect, the UCT in some areas is smaller than the BDd_c_ when the feed rate increases to 5.46 µm/z (Figure 10d). Therefore, the machined surface quality of the microgroove is improved, and only a small number of brittle fracture points appear on the surface. It is the same as the simulated results in Figure 9d, where ductile mode cutting occurs in the local area. Through the above results and discussion, it can be seen that the simulated results are in good agreement with the experimental results, and it is confirmed that the micro scratch defect could change the cutting mode by reducing the UCT.

**Figure 11:**
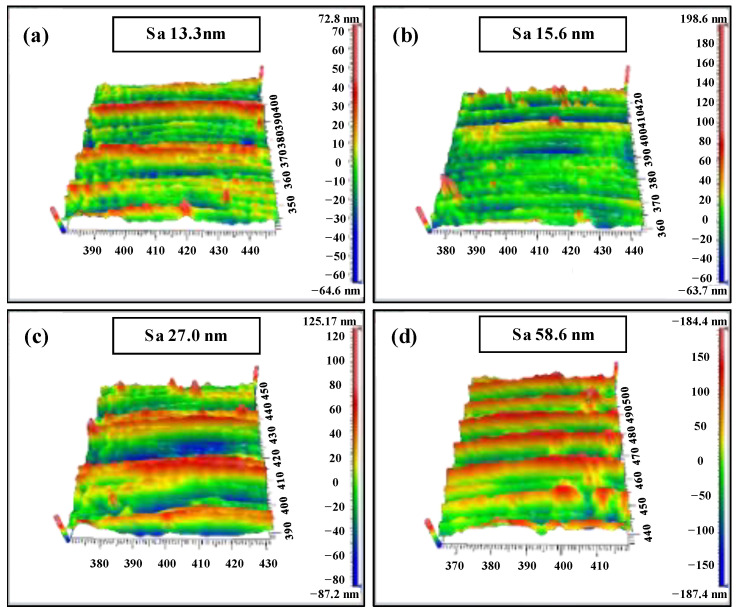
(Scratched surface) Three-dimensional morphological characteristics of experimentally processed microgroove with different feed rates per tooth: (**a**) *f*_t_ = 0.27 µm/z, *f*_t_/*r*_e_ = 0.15; (**b**) *f*_t_ = 0.46 µm/z, *f*_t_/*r*_e_ = 0.25; (**c**) *f*_t_ = 1.37 µm/z, *f*_t_/*r*_e_ = 0.75; (**d**) *f*_t_ = 5.46 µm/z, *f*_t_/*r*_e_ = 3.0.

To further demonstrate the effect of the micro scratch defect on cutting force and surface topography, the white light interferometer was applied to measure the 3D morphology of the machined surface, as shown in Figure 11. Compared with the results in Figure 6a,b, it can be seen from Figure 11a,b that the roughness of the machined surface is higher with the same process parameters, which further proves that the scratch defect will enhance the size effect in the plough cutting mode. It induces a more serious ploughing phenomenon, which is in good agreement with the results in Figure 10a,b. Compared with the results in Figure 6c, the surface roughness increases as shown in Figure 11c under a feed rate of 1.37 µm/z, up to 27 nm. Because the UCT reduces due to the scratch defect, the ploughing effect occurs in the local area of the surface. However, compared with the results in Figure 6d, the machined surface quality of the microgroove is improved and the surface roughness is reduced by 50.8 nm when the feed rate increases to 5.46 µm/z (Figure 11d). It indicates that the UCT in this area is smaller than the BDd_c_ due to the scratch defect. From the analysis above, it can be concluded that the machined surface quality is deteriorated by the micro-defects when the UCT is smaller. On the contrary, the brittle mode in the local area can be transformed into the ductile mode, resulting in the improved surface quality.

The above studies are related to the effect of specific scratch defects on cutting force and machined surface quality involved in the ball-end milling repairing of flawed KDP crystal surfaces. Future research may make efforts to study the effect of micro-defects with different types and sizes on the micro-milling repair processes. It is a challenge and future development direction to promote the recycling of optical components and reveal the effect of micro-defects on the resistance to laser damage after the crystal components are repaired.

## 5. Conclusions

In this work, a 3D simulation model of the micro-milling process for KDP crystals is established based on the proposed constitutive model considering the anisotropic mechanical property. The FEM and micro-milling experiments were conducted to explore the effect of pre-existing micro-defects on the cutting force and machined surface quality involved in the ball-end milling repairing of flawed KDP crystal surfaces. Combined with the simulations and experimental results, the conclusions are obtained as follows:

1. A 3D simulation model of the micro-milling process for KDP crystals considering the anisotropic mechanical property was established, which can be used to study and explain the change of the cutting force and machined surface quality in the micro-milling repair process of KDP crystals.
2. The pre-existing micro-defects can not only affect the amplitude and fluctuation tendency of cutting force in the micro-milling repair process of KDP crystals, but also affect the machined surface topography.
3. The pre-existing micro-defects are capable of reducing the UCT, resulting in more serious relaxation and oscillation of the cutting force when the UCT is smaller, while the cutting force curve becomes smoother and approximates an ideal sinusoidal function.
4. When the UCT is small (less than 375 nm), the pre-existing micro-defects will deteriorate the machined surface quality by reducing the UCT, resulting in more ploughing effects and chips adhered to the crystal surfaces. On the contrary, brittle mode cutting in the local area could be transformed into ductile mode cutting. The machined surface quality could be correspondingly improved.

This work can provide the theoretical basis and technology guidance for determining the processing parameters for the effective repairing and improvement of anti-laser damage ability of KDP functional crystals applied in high-power laser systems.

## Figures and Tables

**Table 1:** Yield stress ratios of KDP crystals.

*R*_11_	*R*_22_	*R*_33_	*R*_12_	*R*_13_	*R*_23_
1	1.2	1.2	0.63	0.63	0.63

**Table 2:** The material parameters in the micro-milling simulation model of KDP crystals [32].

Specific Heat (m^2^/s^2^K)	Density (kg/m^3^)	Coefficient of Thermal Expansion (10^−5^K^−1^)			Thermal Conductivity (kg·m/s^3^K)		
		β_x_	β_y_	β_z_	λ_x_	λ_y_	λ_z_
730	2.344 × 10^3^	1.6	1.6	2.9	2.0	2.0	3.0

**Table 3:** Geometric parameters of the micro-milling tool.

Diameter (mm)	Length of Cut (mm)	Overall Length (mm)	Neck Taper Angle (°)	Shank Diameter (mm)	Effective Length (mm)
0.5	0.38	50	15	4	1.5

**Table 4:** The material properties of the CBN ball-end milling cutter [33,34].

Density (g/cm^3^)	Poisson’s Ratio	Young’s Modulus (GPa)
3.48	0.11	720

**Table 5:** Cutting parameters applied in the micro-milling repair process of KDP crystals.

	Test No.	Spindle Speed (r/min)	Depth of Cut (µm)	Feed Rate (µm/Tooth)	Tool Radius (mm)
Microgroove	1	50,000	4	0.27	0.25
	2			0.46	
	3			1.37	
	4			5.46	
Scratch defect	5		1	0.6	0.15

## Data Availability

Not applicable.
